# Exosome and Microvesicle-Enriched Fractions Isolated from Mesenchymal Stem Cells by Gradient Separation Showed Different Molecular Signatures and Functions on Renal Tubular Epithelial Cells

**DOI:** 10.1007/s12015-016-9713-1

**Published:** 2017-01-09

**Authors:** Federica Collino, Margherita Pomatto, Stefania Bruno, Rafael Soares Lindoso, Marta Tapparo, Wen Sicheng, Peter Quesenberry, Giovanni Camussi

**Affiliations:** 1 Carlos Chagas Filho Institute of Biophysics, Federal University of Rio de Janeiro, Rio de Janeiro, RJ Brazil; 2 Department of Medical Sciences and 2i3T, University of Torino, Torino, Italy; 3 Department of Molecular Biotechnology and Healthy Science, Molecular Biotechnology Center, University of Torino, Torino, Italy; 4 Division of Hematology/Oncology, Rhode Island Hospital, Brown University, Providence, RI USA

**Keywords:** Extracellular vesicles, Mesenchymal stem cells, Exosomes, Microvesicles, Acute kidney injury, Kidney regeneration

## Abstract

Several studies have suggested that extracellular vesicles (EVs) released from mesenchymal stem cells (MSCs) may mediate MSC paracrine action on kidney regeneration. This activity has been, at least in part, ascribed to the transfer of proteins/transcription factors and different RNA species. Information on the RNA/protein content of different MSC EV subpopulations and the correlation with their biological activity is currently incomplete. The aim of this study was to evaluate the molecular composition and the functional properties on renal target cells of MSC EV sub-populations separated by gradient floatation. The results demonstrated heterogeneity in quantity and composition of MSC EVs. Two peaks of diameter were observed (90–110 and 170–190 nm). The distribution of exosomal markers and miRNAs evaluated in the twelve gradient fractions showed an enrichment in fractions with a flotation density of 1.08–1.14 g/mL. Based on this observation, we evaluated the biological activity on renal cell proliferation and apoptosis resistance of low (CF1), medium (CF2) and high (CF3) floatation density fractions. EVs derived from all fractions, were internalized by renal cells, CF1 and CF2 but not CF3 fraction stimulated significant cell proliferation. CF2 also inhibited apoptosis on renal tubular cells submitted to ischemia-reperfusion injury. Comparative miRNomic and proteomic profiles reveal a cluster of miRNAs and proteins common to all three fractions and an enrichment of selected molecules related to renal regeneration in CF2 fraction. In conclusion, the CF2 fraction enriched in exosomal markers was the most active on renal tubular cell proliferation and protection from apoptosis.

**Electronic supplementary material:**

The online version of this article (doi:10.1007/s12015-016-9713-1) contains supplementary material, which is available to authorized users.

## Introduction

Extracellular vesicles (EVs) are small extracellular membrane fragments heterogeneous for their origin, dimensions and content. EVs are mainly composed by exosomes, small homogeneous vesicles (50–150 nm) formed from the endosomal cell compartment, and by microvesicles, with a more heterogeneous dimension profile, produced by the direct extrusion of the cell plasma membrane. Ratajczak et al. [1] first demonstrated that embryonic stem cells may release EVs capable to reprogram hematopoietic progenitors. EVs derived from adult stem/progenitor cells have been described to retain features resembling their cells of origin. In particular, EVs derived from mesenchymal stem cells (MSCs) can mediate the paracrine effects of MSCs in different models of tissue regeneration [2–5]. Selective molecules such as proteins, RNAs and small non coding RNAs are compartmentalized inside EVs [1, 6–10], and may be transferred to target cells via EVs. Numerous studies reported the heterogeneity in EV populations. Exosomes showed the presence of subsets of cellular proteins, such as the tetraspanin family members (CD63, CD81 and CD9), heat-shock proteins [11], and endosomal sorting complexes proteins, such as Alix and TSG101 [12]. Moreover, specific subsets of small RNAs have been described as selectively incorporated in different EV populations [13, 14]. Functional studies have been usually performed using a heterogeneous EV combination or selective sub-fractions [15, 16]. Numerous protocols to isolate EV sub-populations have been described [17], but to identify a method to separate a pure population still remains challenging [18]. EVs from MSCs (MSC EVs) have been extensively used for the treatment of acute kidney injury (AKI), showing regenerative properties. These were ascribed, at least in part, to the transfer of RNA species, such as mRNAs and miRNAs [2, 8, 19, 20]. Comprehensive information on the RNA/protein content of different subpopulation of MSC EVs is currently incomplete.

The aim of this study was to analyze the composition and activity of different EV sub-populations released from MSCs, defining the molecular profile associated with their activity on renal tubular epithelial cells. For this purpose, we fractioned by density gradient the conditioned medium (CM) of MSCs and investigated the different EV subpopulations obtained. Moreover, we combined different gradient fractions (CFs), on the bases of differential expression of exosomal markers and density, and then we characterized their biological activity on renal target cells. The signaling pathway (s) associated with the protein and miRNA cargo content of different EV subpopulations have also been investigated in relation with their biological activity.

## Materials and Methods

### Cell Culture

Bone marrow MSCs were purchased by Lonza (Basel, Switzerland) and cultured as previously described [2]. Cells were used within the seven passages. MSCs characterization was performed by cytofluorimetric analysis for the expression of the typical mesenchymal markers as described [2].

Murine tubular epithelial cells (mTEC) were isolated from the kidneys of healthy C57 mice. Cells were cultured and characterized as previously described by Bruno et al. [2]. mTEC were positive for classical epithelial markers such as: cytokeratin, actin, alkaline phosphatase, aminopeptidase A, and megalin, and negative for CD45, von Willebrand factor, desmin and nephrin.

### Preparation of Extracellular Vesicles-Containing Conditioned Medium

For the preparation of the conditioned medium (CM), MSCs (passage 3–7) were cultured in the presence of their expansion medium until 80% of confluence. Conditioned medium was obtained from supernatants of 8.95 ± 0.46 × 10^3^/cm^2^ MSCs maintained in RPMI medium supplemented with 0.1% BSA for 16 h. The viability of MSCs after starvation was about 86 ± 0.5% as detected by the Muse® Count &Viability Assay Kit (CTRL normal medium 88,9% of vitality) (Millipore, MA, USA). Supernatant was first centrifuged at 1500 g for 20 min, to remove debris and apoptotic bodies and then concentrated at 4 °C, approximately 200-fold, using ultrafiltration units (Amicon Ultra-PL 3, Millipore) with a 3 kDa molecular weight cut-off as previous described [21]. After the concentration CM-containing EVs in 1% dimethyl sulfoxide was kept at −80 °C until use.

### Density Gradient Separation of EVs

A discontinuous iodixanol gradient was prepared as described by Tauro et al. [22]. Solutions of 5, 10, 20 and 40% iodixanol were obtained by mixing an OptiPrepTM (60% *w*/*v* aqueous iodixanol solution) (Sigma-Aldrich, St. Louis, MO) with the appropriate amounts of homogenization buffer (0.25 M sucrose/ 10 mM EDTA/ 10 mMTris-HCL, pH 7.4). The gradient was formed by layering 3 ml of 40%, 3 ml of 20%, 3 ml of 10% and 2.5 ml of 5% solutions in a 13 ml open top polyallomer tube (Beckman Coulter). 500 μl of CM-containing EVs (cCM-EVs) were overlaid onto the top of the gradient [22] or at the bottom [23] and centrifuged for 18 h at 100,000 g at 4 °C (SW 40 Ti rotor, Beckman Coulter Optima L-90 K ultracentrifuge, Indianapolis, IN). Preliminary experiments demonstrated the same vesicles distribution using the protocols listened above (not shown) and the loading from the top of the gradient [22] was used in the following experiments.

Twelve gradient fractions of 1 ml, were collected from the top of the gradient, diluted with 10 ml PBS for washing and centrifuged at 100,000 g for 2 h at 4 °C. The pellets were re-suspended in 100 μl PBS or medium based on the following use. The density of each fraction was measured by weighing a fixed volume [16]. In some experiments, fractions were divided into three groups (combined fractions, CFs), based on their density and on the expression of specific surface markers (Fractions 1–4 low density, CF1; Fractions 5–8, medium density CF2; Fractions 9–12, high density CF3).

### EV Incorporation

To trace EVs by fluorescence microscopy, MSCs were labeled with Vybrant Cell Tracers Dil and Syto-RNA (Life Technologies, Carlsbad, CA) as previous described [20]. EVs obtained from labeled cells were concentrated and subjected to gradient separation as described above. For EV incorporation, mTEC were plated in 24-well plated and treated with different doses of labeled cCM-EVs (50,000, 150,000, 300,000 or 600,000 EVs/cell) for 24 h. Quantitative analysis of the EV uptake was conducted by FACS. To determine the incorporation of each fraction, mTEC were seeded into 24-well plates and incubated with labeled EVs (150,000 EVs/cell) from the different combined fractions for 24 h. The up-take of EVs was analyzed by microscope analysis using the ApoTome system (Carl Zeiss, Oberkochen, Germany). Hoechst 33,258 dye (Sigma-Aldrich) was added for nuclear staining.

### In Vitro Models

mTEC were seeded at 1500 cells/well into 96-well plates and cultured in serum free low-glucose DMEM in the absence (vehicle, CTR-) or presence of EVs (1 × 10^7^ EVs/ml, 1 × 10^8^ EVs/ml or 1 × 10^9^ EVs/ml). Cells maintained in low-glucose DMEM (Sigma-Aldrich) plus 10% FCS were used as positive control (CTR+). In selected experiments, to mimic the ischemia damage on renal cells, mTEC cultured in serum free DMEM were placed in hypoxic chambers with 1% O_2_ for 48 h. The re-oxygenation step was conducted for 24 h, in the absence (vehicle, HY/CTR-) or presence of EVs (1 × 10^7^ EVs/ml). DMEM plus 10% FCS (HY/CTR+) or EGF (10 ng/ml, Sigma) (HY/EGF) in the re-oxygenation step were used as controls. For cell proliferation, DNA synthesis was detected as incorporation of 5-bromo-2′-deoxy-uridine (BrdU) into the cellular DNA at 48 h (Roche Applied Science, Mannheim, Germany). Apoptosis/necrosis was measured by Muse™ Caspase-3/7 Kit (Millipore) following the instructions. Percentage for live, apoptotic, and necrotic cells was measured.

### EV Characterization

Gradient isolated EVs were analyzed by nanoparticle tracking analysis (NTA), using the NanoSight LM10 system (NanoSight Ltd., Amesbury, UK), equipped with a 405 nm laser and with the NTA 2.3 analytic software, to define their dimension and profile. Camera levels were for all the acquisition at 16 and for each sample, five videos of 30 s duration were recorded. Briefly, cCM-EVs or gradient-separated EVs were diluted (1: 1000 and 1: 10, respectively) in 1 ml vesicle-free physiologic solution (Fresenius Kabi, Runcorn, UK). NTA post-acquisition settings were optimized and maintained constant among fractions, and each video was then analyzed to measure EV mean, distribution and concentration.

EVs from the twelve fractions were characterized by cytofluorimetric analysis using the Guava easyCyte Flow Cytometer (Millipore) with InCyte software. The following FITC or APC conjugated antibodies were used: CD107, CD81 and CD63. FITC or APC mouse non-immune isotypic IgG (Miltenyi Biotec, Bergisch Gladbach, Germany) were used as controls as previously described [20]. Briefly, immediately after labelling for 15 min at 4 °C with antibodies, EVs (1.5 × 10^8^ particles) diluted 1 to 3 were acquired.

### Western Blot Analysis

For protein analysis, EVs from different fractions and MSCs were lysed at 4 °C for 30 min in RIPA buffer (20 nM Tris-HCl, 150 nM NaCl, 1% deoxycholate, 0.1% SDS, 1% Triton X-100, pH 7.8) supplemented with protease and phosphatase inhibitors cocktail (Sigma-Aldrich). Protein content in EV fractions were quantified by BCA Protein Assay Kit (Pierce, Thermo Fisher Scientific, Waltham, MA). Ten μg of proteins were then separated by 4% to 15% gradient sodium dodecyl sulfate–polyacrylamide gel electrophoresis. The proteins were transferred onto a PVDF membrane by the iBlot™ Dry Blotting System (Life Technology) and then immunoblotted with the following antibodies: CD63 and ANXA2 (Santa Cruz Biotechnology, Santa Cruz CA), HLA1 (Abcam, Cambridge, United Kingdom), CD29 (Thermo Fisher Scientific) and Integrin alpha-5 (Millipore). The protein bands were visualized using a ChemiDoc™ XRS + (BioRad) with an enhanced chemiluminescence detection kit (ECL) (GE healthcare, Amersham, Buckinghamshire, UK).

### Electron Microscopy

Transmission electron microscopy of EVs was performed by loading EVs from different CF onto 200 mesh nickel formvar carbon coated grids (Electron Microscopy Science, Hatfield, PA) for 20 min. EVs were then fixed with a solution containing 2.5% glutaraldehyde and 2% sucrose and after repeated washings in distilled water, samples were negatively stained with NanoVan (Nanoprobes, Yaphank, NK, USA) and examined by Jeol JEM 1010 electron microscope.

### RNA Isolation

Total RNA was isolated from gradient separated EVs using the mirVana RNA isolation kit (Applied Biosystem) according to the manufacturer’s protocol. RNA from all the twelve fractions or from the three CFs was quantified (Nanodrop ND-1000, Wilmington DE) and the small RNA composition of different CFs was assessed by capillary electrophoresis on an Agilent 2100 Bioanalyzer using the small RNAs kit (Agilent Technologies, Inc., Santa Clara, CA).

### miRNA Screening

Purified CF-derived EVs isolated from three different MSCs preparations were analyzed for their miRNA content by quantitative real time (qRT) PCR using the Applied Biosystems TaqManH MicroRNA Assay Human Panel Early Access kit (Life Technologies), able to profile 754 human mature miRNAs by sequential steps of reverse transcription (Megaplex RT Pools; Life Technologies) using an Applied Biosystems 7900H qRT-PCR instrument as previously described [20]. Briefly, single stranded cDNA was generated from total RNA sample (80 ng) by reverse transcription using a mixture of looped primers (Megaplex RT kit, Life Technologies) following manufacturer’s protocol. The pre-amplification reaction for each sample was performed using a TaqMan® PreAmp Master Mix 2X (Life Technologies) mixed with specific Megaplex™ PreAmp Primers (10X) (Life Technologies). Pre-amplified products were then diluted, loaded in the TaqMan MicroRNA Array and qRT-PCR experiments were performed.

Raw Ct values, automatic baseline and threshold were calculated using the SDS software version 2.3. Comparison of miRNA expression was conducted using the Expression Suite software (Life Technologies). Fold change (Rq) in miRNA expression among the three fractions was calculated as 2^-ΔΔCt^ using one of the fraction (CF2) as control and normalizing the data using global normalization [24]. Confirmation of the expression of specific miRNAs in the three fractions was conducted using the miScript SYBR Green PCR Kit (Qiagen, Valencia, CA, USA). Briefly, 50 ng of input RNA were reverse transcribed using the miScript Reverse Transcription Kit and the cDNA was then used to detect and quantify miRNAs of interest. Experiments were run in triplicate using 3 ng of cDNA for each reaction as described by the manufacturer’s protocol (Qiagen). The following miRNAs were screened in all the CF: miR-100, miR-21, miR-24, miR-214, miR-34a, miR-127, miR-30c, miR-29a, miR-125b, miR-10b, let-7c, miR-99a, miR-17 and miR-20a.

Analysis of miR-451 distribution in the twelve fractions was carried on using the miRCURY LNA™ Universal RT microRNA PCR kit (Exiqon, A/S, Vedbaek, Denmark). Fifty pg of reverse transcription reaction products were then combined with SYBR Green Master Mix (Exiqon) and LNA™ PCR primer mix and analyzed as described by the manufacturer’s protocol. All the qRT-PCR data were normalized using the UniSp6 and the UniSp2 RNA Spike-in templates respectively as cDNA synthesis and RNA extraction controls (Exiqon).

### Protein Array

Purified CF-derived EVs isolated from different MSC preparations were lysed in 2× Cell Lysis Buffer (RayBiotech, Inc., GA), and 15 μg of EV proteins from all the CFs, were used for RayBio Label-based (L-Series) Human Antibody Array 1000 (RayBiotech) according to the manufacturer instructions. The arrays were performed in duplicates, using a pull of EVs derived from two different MSC preparations/array. The array provides detection of 1000 proteins.

Data analysis was conducted after background signal subtraction and normalization to positive controls (Mean background +4 standard deviations, accuracy ≈ 99%). Comparison of signal intensities among array images was used to define relative differences in expression levels of each protein among the CF fractions. Differential expression analysis was conducted using CF2 as reference fraction. Proteins were considered co-expressed when they showed a Fold change (FC) = 0.65 ≤ FC ≤ 1.5 in all sample tested. Differential expression among CFs was considered when FC distribution: <0.65 or >1.5 in all the sample tested.

### Pathway and Gene Ontology Analysis of EV Content

For CF2 miRNA target prediction and biological pathway enrichment analysis, the web-based program DIANA-mirPath [25] was used. The algorithm microT-CDS was chosen to predict EV-derived miRNA targets using default threshold (microT = 0.8). Only biological pathways showing *P value < 0.01* to all known Kyoto Encyclopedia of Genes and Genomes (KEGG) pathways were considered as significantly enriched.

For protein class analysis and pathway classification Panther classification system was used (http://pantherdb.org/). Gene Ontology (GO) analysis was conducted using David functional annotation tool (http://david-d.ncifcrf.gov.). Functional sorting of the proteins differentially expressed between CF2 and the other fractions was done using Funrich analysis tool [26].

### Statistical Analysis

Data were analyzed using the GraphPad Prism 6.0 Demo program. Statistical analyses were conducted using One-way ANOVA with Dunnett’s or Turkey’s post-tests, where appropriated. Statistical significance was established at *P < 0.05*.

## Results

### Heterogeneity of EVs Released from MSCs.

**Fig. 1:**
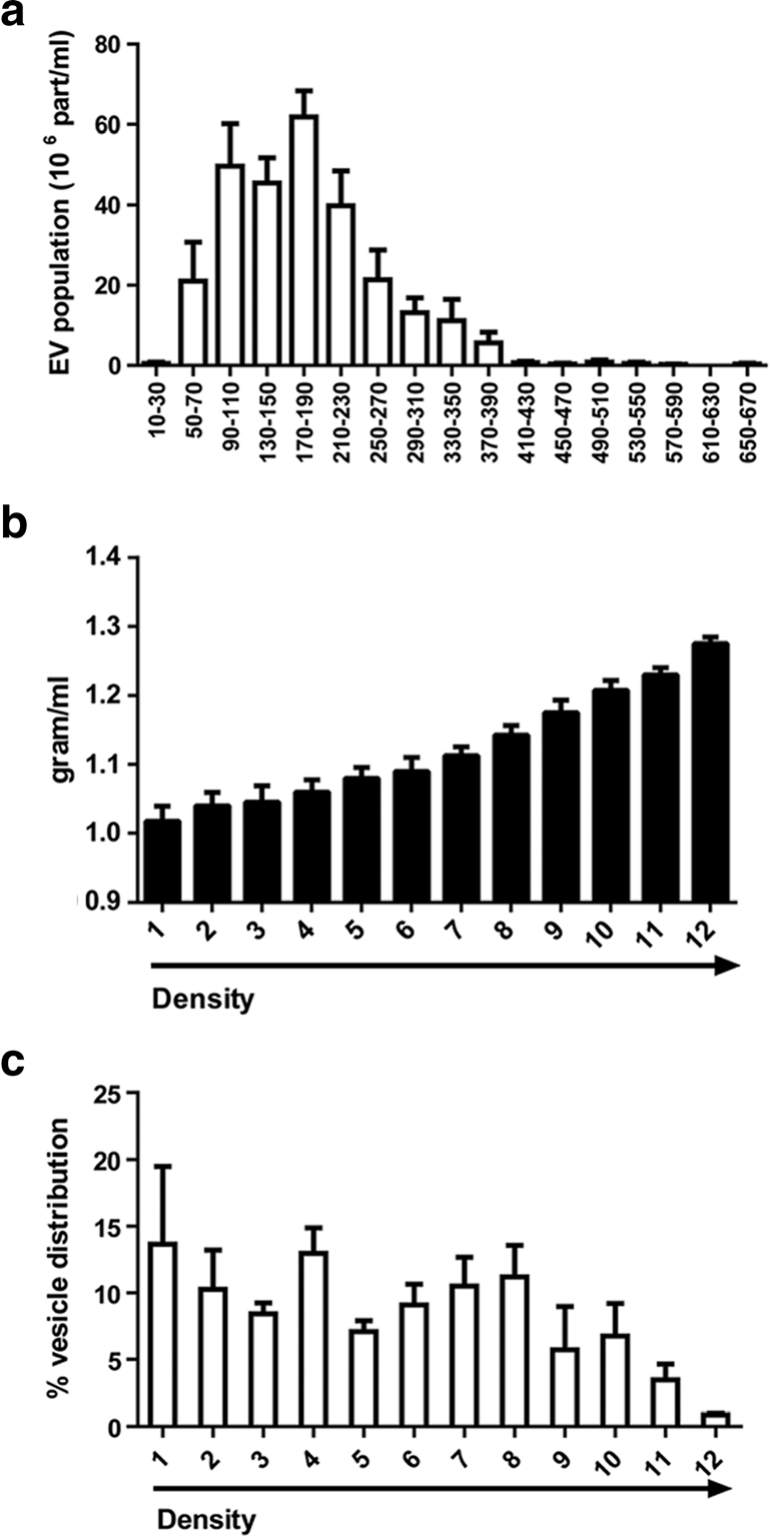
Characterization of EVs isolated from the conditioned medium (CM) of human MSCs and subjected to OptiPrep gradient separation. **a** Nanoparticle Tracking Analysis (NTA) profiles of particle size and distribution of EVs present in the concentrated CM of MSCs (cCM-EVs). cCM-EVs represent a extracellular vesicle population containing exosomes and microvesicles. Two peaks around 90–110 and 170–190 nm were detected. **b** Weight of the twelve different EV fractions generated by gradient separation of cCM-EVs. **c** NTA analysis of the percentage of EVs distributed in different fractions after gradient separation. Data reported are mean ± SEM of four different experiments

To isolate a total EV population from MSCs, we pre-purified their CM as described in Material and Methods. The CM was then subjected to concentration and the number of isolated EVs was counted by NTA. We observed that the mean number of EVs present in the concentrated CM was around 12,750 ± 3187 particles/cell. The cCM-EVs represented a mix population with different diameter, ranging from 50 to 390 nm (Fig. 1a). Two peaks of diameter were defined around 90–110 and 170–190 nm (Fig. 1a) with a mean diameter in the total population of 136 ± 16 nm and a mode of 119 ± 21 nm. The identification of a heterogeneous population prompts us to define its molecular content and activity. For this purpose, we fractionated the cCM-EVs by density gradient separation, isolating twelve different EV fractions. Density of each fraction was measured by weighing a fixed volume (Fig. 1b) [16]. The analysis of EV distribution by NTA, showed an enrichment of vesicles in fractions 1, 4 and 8 (Fig. 1c).

### Characterization of EVs in Different Fractions.

**Fig. 2:**
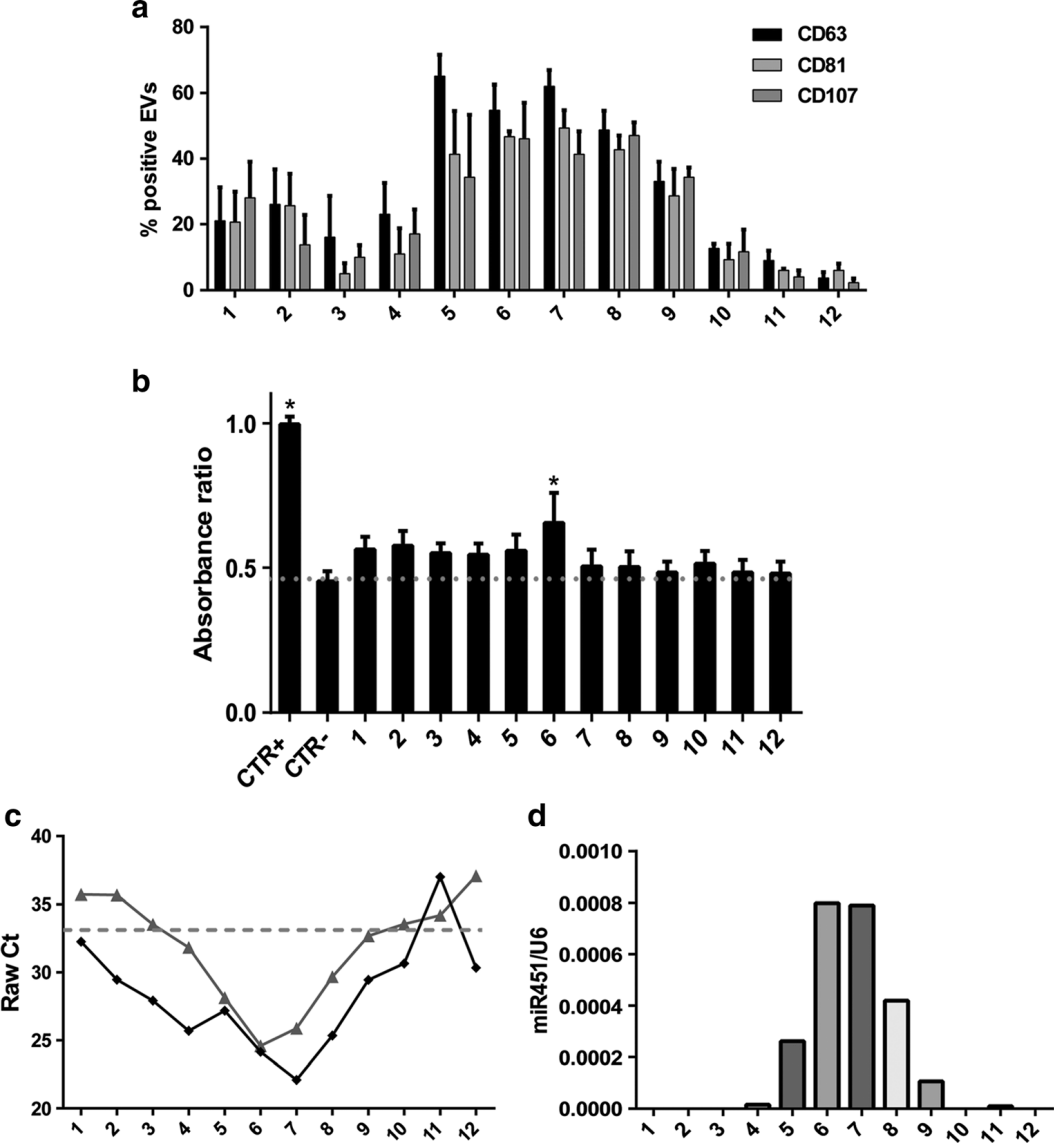
Characterization of specific exosome markers and activity of the twelve EV fractions isolated by gradient separation. **a** FACS analysis of the expression of the tetraspanin members, CD63 and CD81 and the lysosomal-associated protein, CD107 in different EV fractions. The exosome markers showed a relevant co-localization in fractions 5–8, characterized by a flotation density of 1.08–1.14 g/mL. **b** Evaluation of the effects of different EV fractions on mTEC proliferation after 48 h of stimulation, assessed by BrdU up-take (1 × 10^7^ EVs/ml) in respect to control cells (CTR-, DMEM no FCS). Cells cultured in DMEM plus 10% FCS were used as positive control (CTR + =1). Data are expressed as Ratio means ±SEM. ANOVA with Dunnett’s multicomparison test. *P < 0.05 vs CTR-. **c** Representive qRT-PCR expression of miR-21 and miR-451 in the twelve fractions. Raw data analysis of miR-21() shows its expression in almost all the EV fractions. On the contrary, miR-451 () shows selective compartmentalization in the central fractions. **d** Representative qRT-PCR analysis showing the relative quantity of miR-451 in respect to the synthetic spike-in (UniSp6) used as normalizer. Normalized data showed the distribution of miR-451 inside the exosome-enriched EV fractions. Three experiments were conducted with similar results

By FACS analysis the expression of CD63 endosome-derived exosome marker was mainly observed in fractions 5–8, with a flotation density of 1.08–1.14 g/mL (Fig. 2a). The other tetraspanin family member, CD81 and the lysosomal-associated membrane protein 1, CD107, showed almost the same sinusoidal pattern (Fig. 2a). As shown in Fig. 2b, the biological activity on mTEC is widely distributed in different fractions, except for the densest fractions, but is significantly higher in fraction 6.

Total RNA was extracted from all the twelve fractions and the major concentration of RNA was isolated from fractions 5, 8, 9 and 11 (not shown). Since it has been suggested a selective export of specific miRNAs from different cell compartments [10], we evaluated the distribution of a group of miRNAs that we previously described in MSC-derived EVs [8]. miR-21 was expressed in almost all the EV fractions. However, as shown in Fig. 2c, the Raw Ct of miR-21 indicated its enrichment in the central fractions (Fig. 2c). Similar enrichment pattern was observed for miR-100, −99a and −24 (data not shown). Conversely, miR-451 was detected only in central fractions enriched in the tetraspanin family markers (Fig. 2C) and this distribution was compatible with the exosomal derivation of this miRNA. To exclude the possibility of confounding technical factors on the obtained results, we performed miR-451 quantification adding a synthetic spike-in (UniSp6) during the cDNA synthesis. We obtained the same results, demonstrating the effective compartmentalization of miR-451 mainly in the CD63 positive fractions (Fig. 2d).

### Biological Effect and Characterization of EV Combined Fractions

**Fig. 3:**
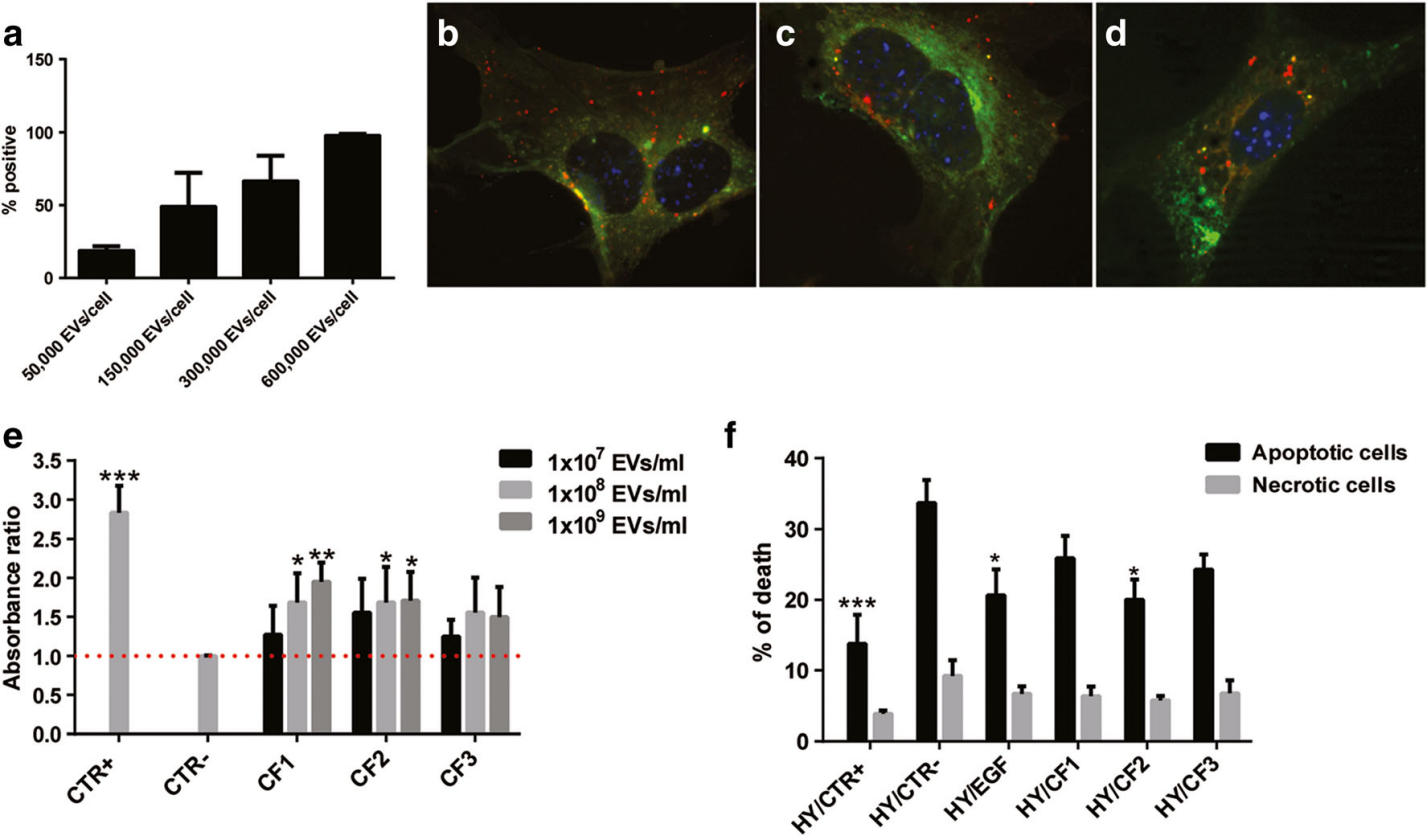
Activity of the combined gradient fractions on tubular cell proliferation and apoptosis. **a** Quantitative FACS analysis of the up-take of labelled cCM-EVs by mTEC. A dose dependent up-take of cCM-EVs (50,000–600,000 EVs/cell) was observed after incubation for 24 h. **b-d** Representative micrograph of the internalization of EVs from CF1 (**b**), CF2 (**c**) and CF3 (**d**) by mTEC after 24 h, observed by confocal microscopy. EVs were collected from MSCs double-stained with Syto-RNA (green) and Vybrant Dil (*red*). Three experiments were performed with similar results. Nuclei were counterstained with Hoechst dye. Original magnification: ×630. (E-F) Evaluation of the effects of the combined fractions on mTEC proliferation (**e**) and protection from apoptosis (**f**). **e** Absorbance ratio of the BrdU up-take by mTEC incubated for 48 h with EVs from different CFs (1 × 10^7^ EVs/ml, 1 × 10^8^ EVs/ml or 1 × 10^9^ EVs/ml) in respect to control cells (CTR-, DMEM no FCS). Cells cultured in DMEM plus 10% FCS were used as positive control (CTR+). **f** Cell death analysis on mTEC subjected to hypoxia/reperfusion was measured by Muse™ Caspase-3/7 Kit. Cells were subjected to hypoxia for 48 h, then EVs from CFs were added during the 24 h of reperfusion (1 × 10^7^ EVs/ml). Cells maintained in the absence of serum were used as negative control (HY/CTR-). Cells cultured in DMEM plus 10% FCS in the reperfusion phase were used as positive control (HY/CTR+). Black bars indicate apoptosis and grey bars represent necrosis. Data are expressed as means ±SEM. ANOVA with Dunnett’s multicomparison test. *P < 0.05, **P < 0.01, ***P < 0.001 vs CTR- or HYP/CTR-, respectively

In order to pair the biological activity with the molecular content of the EV subpopulations, we combined the gradient fractions into three groups (Fractions 1–4, low density CF1; Fractions 5–8, medium density CF2; Fractions 9–12, high density CF3), based on their density and on the expression of exosome surface markers and miRNAs. We first analyzed the up-take of labelled cCM-EVs by mTEC. Figure 3a shows a dose dependent up-take of cCM-EVs. We then tested the uptake of EVs from the different fractions using confocal microscopy (Fig. 3b-d). Using EVs labelled with Vybrant Dil and with Syto-RNA, we observed the concomitant presence within the cells of the two dyes, suggesting the EV delivery of RNAs inside mTEC.

To discriminate the effects of the different EV fractions on cell proliferation and apoptosis, in vitro experiments were performed. Despite all EV fractions slightly promoted cell proliferation on mTEC, only the low density CF1 and medium density CF2 fractions induced a statistically significant proliferation at 1 × 10^8^ and 1 × 10^9^ particles/ml concentration (Fig. 3e). We next evaluated the effects of the combined fractions in a hypoxia/reperfusion model that mimics renal tubule damage during the ischemia reperfusion injury (IRI). For this purpose, mTEC were treated with CFs during the re-oxygenation phase of IRI. Only the CF2 medium-density vesicles together with the EGF treatment during the reperfusion phase were anti-apoptotic (Fig. 3f). On the contrary, CF1 low-density vesicles or CF3 high density EVs were not significantly protective on mTEC submitted to IRI.

**Fig. 4:**
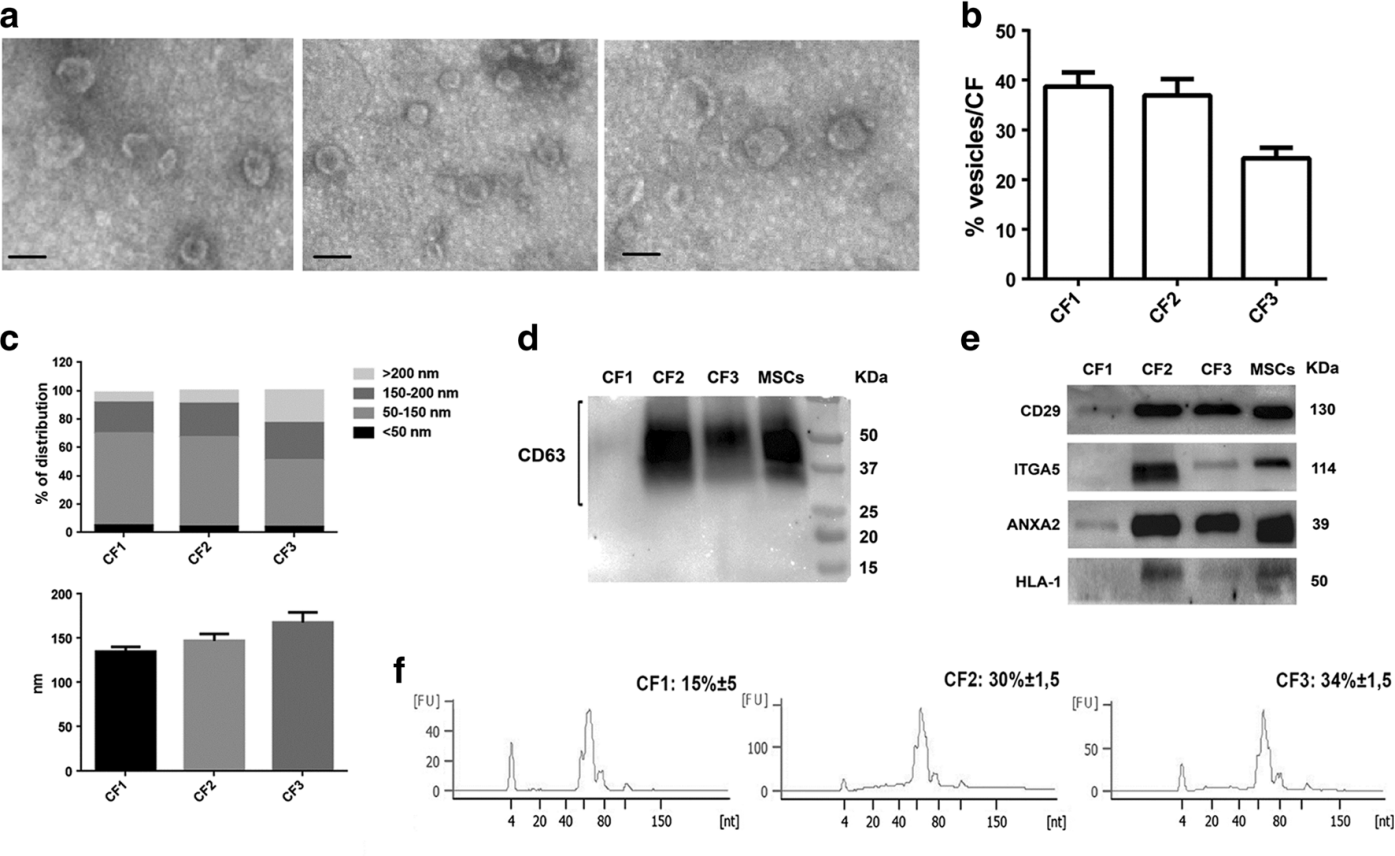
Characterization of morphology, protein surface and miRNA content of combined fraction EVs. **a** Transmission electron microscopy (original magnification × 75,000) of EVs from different CFs. All the CFs contain vesicles with a typical cup-shaped morphology (*scale bar* 100 nm). Particles with a different electron density were observed in the CF3 fraction. **b** Percentage of EVs isolated for each combined fraction detected by NTA. The CF3 resulted the fraction with less EVs in respect to the low-density CF1 and medium-density CF2 fractions. **c** Percentage of size distribution of EVs in different CF fractions measured by NTA (*upper panel*). The mean diameter (nm) of each CF populations was also measured (*lower panel*). Four different gradients were tested in triplicate. (**d-e**) Representative Western blot analysis of CD63 (**d**), integrin β1 (CD29), α5-integrin (ITGA5), annexin A2 (ANXA2) and HLA-class I (**e**) on MSCs and CF derived EVs. Three different experiments were performed with similar results. (**f**) Representative bioanalyzer profile of small RNAs performed on EVs from the CFs, showing a relevant enrichment of total miRNAs in the medium density CF2 and high density CF3 fractions in respect to CF1. Three different samples tested in triplicate with similar results

To define the molecular composition of the EV gradient fractions, accountable for their different biological potential, further characterization studies were conducted. Transmission electron microscopy analysis performed on purified EVs showed their spheroid morphology (Fig. 4a). The size of EVs present in CF1 was more heterogeneous of the CF2 ones. NTA quantification of the particle distribution among the CF demonstrated an enrichment of vesicles in CF1 and CF2 in respect to CF3 fraction (Fig. 4b). As shown in Fig. 4c, NTA measure of the percentage of size distribution indicated that CF1 and CF2 contain smaller EVs (50–150 nm) whereas the CF3 is also enriched in EVs of larger size (>200 nm). The mean diameter of the different populations was respectively: 134.7 ± 19,7 for CF1, 147 ± 27,5 for CF2 and 169 ± 39 for CF3 (Fig. 4c, lower panel).

The expression of some classical vesicular markers (CD63, ANXA2 and CD29) was compared among the three CFs and their cells of origin by Western blot analysis (Fig. 4d-e). The integrin alpha-5 and HLA-1 were also screened in the different fractions (Fig. 4e). CD63 was almost absent in low-density vesicles of CF1, being enriched in the medium-density EVs of CF2 fraction and into a lesser extent in the high-density CF3 fraction (Fig. 4d). The integrin β1 (CD29), showed the same distribution of the tetraspanin CD63, being enriched in CF2 and CF3 and expressed at low levels in CF1 fraction (Fig. 4e). ANXA2, involved in recruitment of miRNAs in EVs, resulted mainly present in fraction CF2 and CF3 (Fig. 4e), supporting the relevant enrichment of miRNAs detected in these two fractions (Fig. 4f). The HLA-class I and α5-integrin (ITGA5) were selectively expressed by the medium-density fraction CF2 (exosome enriched fraction) and almost absent in CF1 low-density fraction (Fig. 4e). Low expression of these two markers was observed in the CF3 high-density fraction (microvesicle enriched fraction).

### miRNA Compartmentalization inside CF Fractions

**Fig. 5:**
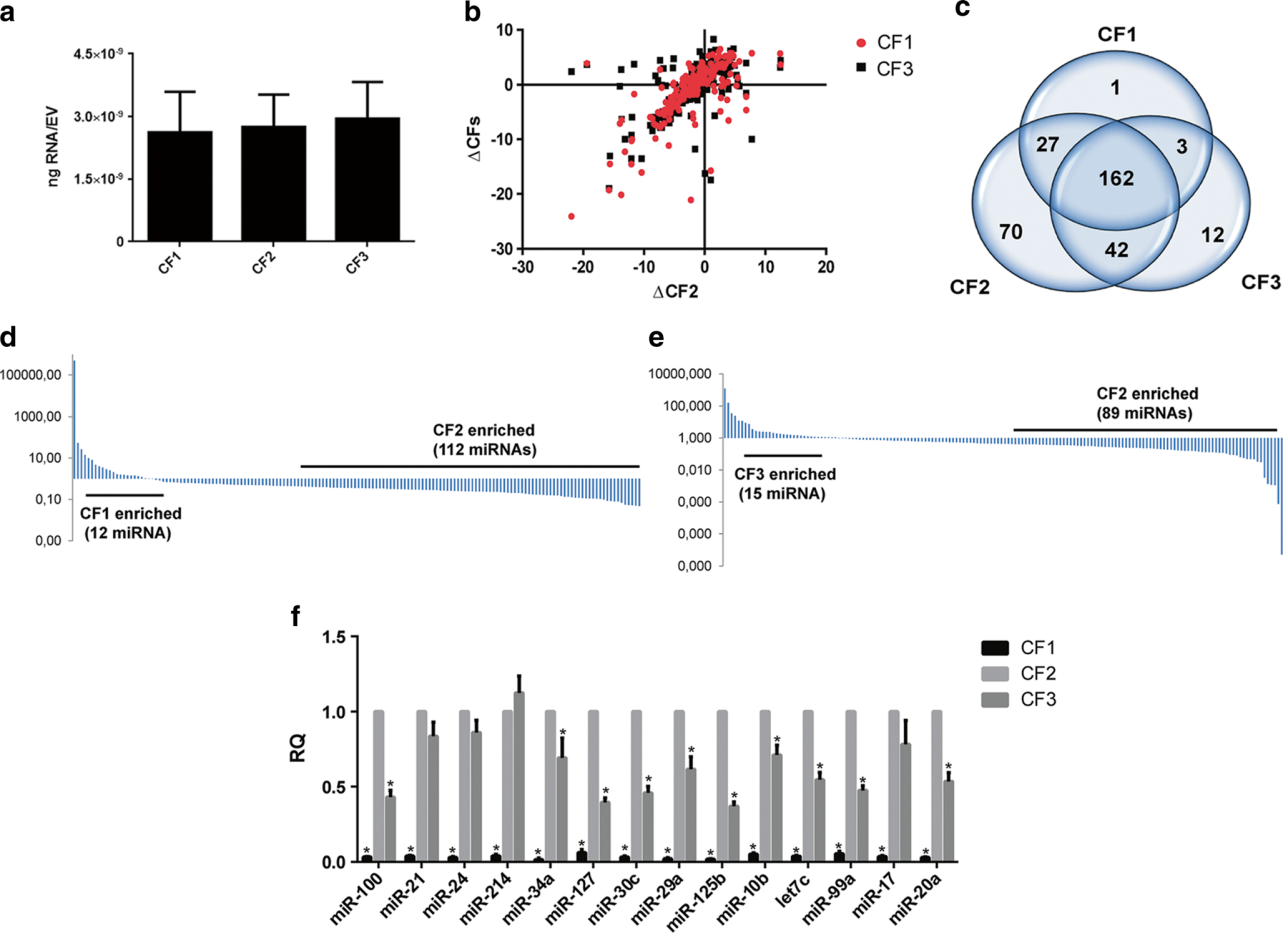
Analysis of miRNA compartmentalization inside the CF fractions. **a** Quantification of the total RNA isolated from the different CFs. Data are expressed as means ±SEM (ng RNA/EV) of four different experiments. No significant differences were observed among the three fractions. (**b-e**) qRT-PCR profile of 754 mature miRNAs in the CF fractions. **b** Scatter plot of normalized Cycle threshold distribution (∆Ct) of all expressed miRNAs (Ct < 40) between CF2 and the other fractions. Pearson correlation was calculated (CF2 vs CF3: 0.50 and vs CF1: 0.69). **c** Venn diagram showing the miRNAs present in all the EV fractions (*n* = 162). A subset of miRNAs was specific of the medium-density CF2 EVs and undetected in the others. Fold change distribution of the co-expressed miRNAs (miRNA intersection) between CF1 and CF2 fractions (**d**) and between CF3 and CF2 fractions (**e**), showing a general enrichment of miRNAs in fraction CF2. (**f**) Validation of the different compartmentalization of specific miRNAs in CFs. The expression of miRNAs enriched in MSC EVs and/or connected with kidney regeneration was analyzed by qRT-PCR. All the miRNAs tested were reduced in CF1, demonstrating less ability of this fraction to compartmentalize miRNAs. The relative quantity of each miRNA (RQ) was measured using the synthetic spike-in (UniSp2) as normalizer. Three different samples tested in triplicate with similar results. Data are expressed as means ±SEM. ANOVA with Dunnett’s multicomparison test. *P < 0.05 vs CF2

RNA was extracted by different CFs and spectrometrically quantified. The bioanalyzer profile showed the enrichment of RNA of the size of miRNAs in the medium-density CF2 and high-density CF3 fractions (Fig. 4f). No relevant differences in the total RNA isolated in respect to the EV quantity among the three CFs was observed (CF1: 2.62 ± 0.97, CF2: 2.75 ± 0.78, CF3: 2.94 ± 0.88 × 10^−9^ ng/particle) (Fig. 5a). The expression of 754 human miRNAs was measured by qRT-PCR. Analyzing the normalized Cycle threshold distribution (∆Ct, based on Median calculation) of all expressed miRNAs (Ct < 40), the medium-density EVs of CF2 fraction showed a low correlation with both CF3 and CF1 EV populations (Pearson correlation: 0.50 and 0.69, respectively) (Fig. 5b).

Using a cut-off ≤35 Ct value in miRNA expression, we found 162 miRNAs present in all the EV fractions (Fig. 5c; Table 1). The analysis showed a selective package of miRNA subsets in medium-density CF2 EV fraction which were undetected in the other fractions (Table 2) (Fig. 5c). Among these miRNAs, we observed 19 miRNA* sequences detected only in the CF2 fraction, as previously described for tumor derived exosome population [14].

**Table 1:**
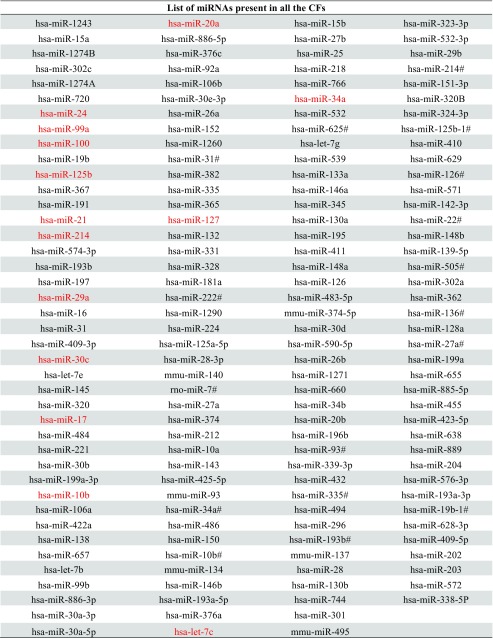
List of the miRNAs detected in all the CF fractions. The miRNAs confirmed by qRT-PCR were labeled in red

**Table 2:** List of the miRNAs selectively detected inside the CF2 fraction

Selective miRNA in medium-density CF2 EVs		
hsa-miR-107	hsa-miR-542-5p	hsa-miR-191#
hsa-miR-122	hsa-miR-545	hsa-miR-206
hsa-miR-135b	hsa-miR-570	hsa-miR-20a#
hsa-miR-190	hsa-miR-579	hsa-miR-223#
hsa-miR-198	hsa-miR-616	hsa-miR-24-1#
hsa-miR-199b	hsa-miR-95	hsa-miR-24-2#
hsa-miR-205	hsa-miR-98	hsa-miR-25#
hsa-miR-211	dme-miR-7	hsa-miR-27b#
hsa-miR-301b	hsa-let-7f-2#	hsa-miR-302d
hsa-miR-302b	hsa-let-7 g#	hsa-miR-361-3p
hsa-miR-32	hsa-miR-106b#	hsa-miR-378
hsa-miR-331-5p	hsa-miR-1179	hsa-miR-380-5p
hsa-miR-34c	hsa-miR-1201	hsa-miR-411#
hsa-miR-369-3p	hsa-miR-1226#	hsa-miR-550
hsa-miR-375	hsa-miR-1227	hsa-miR-580
hsa-miR-381	hsa-miR-1254	hsa-miR-603
hsa-miR-383	hsa-miR-1270	hsa-miR-744#
hsa-miR-449	hsa-miR-1276	hsa-miR-770-5p
hsa-miR-450a	hsa-miR-1291	hsa-miR-941
hsa-miR-492	hsa-miR-130b#	hsa-miR-942
hsa-miR-500	hsa-miR-144#	hsa-miR-944
hsa-miR-502	hsa-miR-15a#	hsa-miR-99a#
hsa-miR-518a-3p	hsa-miR-15b#	
hsa-miR-542-3p	hsa-miR-16-1#	

By analyzing the fold change distribution of the co-expressed miRNAs among all the fractions (miRNA intersection), we observed a relevant enrichment of miRNAs in CF2 fraction containing exosome enriched-EVs. Among the 162 miRNAs expressed by all the fractions, we found that 112 miRNAs were significantly up-regulated in the CF2 fraction in respect to CF1 (Fig. 5d) and only 12 miRNAs were down-regulated. The same trend was observed between CF2 and CF3 (89 miRNAs up-regulated and 15 down-regulated in CF2 in respect to CF3) (Fig. 5e).

In CF2, we detected 106 miRNA families with an enrichment of the following families: miR-10, miR-17, miR-154, miR-30, miR-27, miR-15, let-7, miR-379, miR-26, miR-34 and miR-548 families (not shown).

We confirmed by qRT-PCR the compartmentalization of miRNAs highly enriched in MSC EVs and/or connected with kidney regeneration in the CF2 fraction (miR-100, −21, −24, −214, −34a, −127, −30c, −29a, −125b, −10b, −let-7c, −99a, −17 and miR-20a) [8, 27–32] (Fig. 5f). Some miRNAs, such as miR-21, −24, −214, and −17 were also enriched in the dense fraction CF3, possiblly being a common signature of different EV sub-fractions. Reduced miRNA content was detected in the low-density EV CF1 fraction (Fig. 5f).

Analysis of the pathways over-represented by the predicted targets of enriched/selective CF2 miRNAs was performed with the DIANA mirPath software, as previously described [33]. We detected an enrichment of 14 KEGG biological processes for the CF2 enriched miRNAs and 11 KEGG processed for the CF2 selective miRNAs (*P < 0.01*, FDR corrected) (S1 Table and S2 Table). Sixty-nine miRNAs from the two groups showed strong correlation with the same pathways assembled in metabolic, stem cell associated- and migration/inflammation processes.

**Fig. 6:**
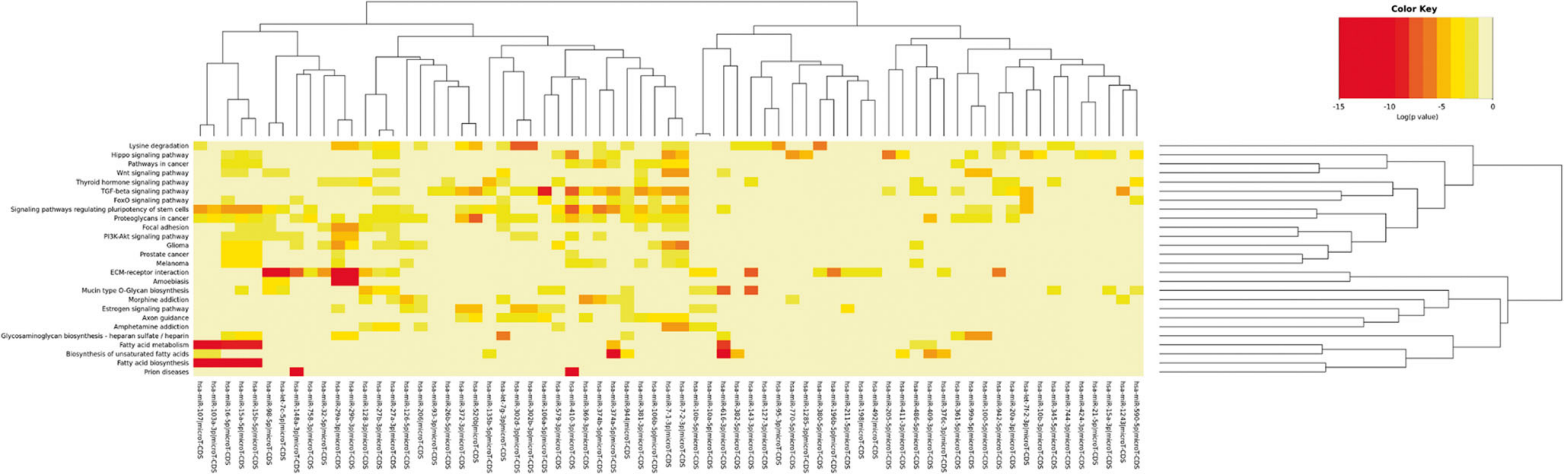
Heatmap representation of the most significantly enriched pathways potentially modulated by the selective/enriched miRNAs in CF2 fraction. Enrichment analysis of the pathways over-represented by the predicted targets of enriched/selective CF2 miRNAs was conducted using the software DIANA mirPath. miRNAs with similar patterns in targeting significant pathways clustered together (69 miRNAs). Strong correlation with the pathways assembled in metabolic, stem cell associated- and migration/inflammation processes was observed. Only pathways targeted by the selected miRNAs with a *P-value < 0,01* (FDR corrected) were considered for the analysis

The following metabolic-related pathways were over-represented: fatty acid biosynthesis and metabolism, biosynthesis of unsaturated fatty acids, mucin type O-glycan biosynthesis and lysine degradation (Table 3). Pathways related to stem cells were: Hippo signaling, Wnt signaling and pluripotent stem cells regulated-pathways (Table 3). Migration-inflammation related processes such as ECM-receptor interaction, TGF-β signaling pathway, glioma and proteoglycans in cancer were also over-represented (Table 3). Heatmap representation of the most significantly enriched pathways potentially modulated by the 69 miRNAs is shown in Fig. 6.

**Table 3:** Biological pathways over-represented by the 69 enriched/selected miRNAs of the medium-density EVs in CF2 fraction (*P < 0,01*; corrected FDR)

KEGG pathway	*p*-value	#genes	#miRNAs
Fatty acid biosynthesis	<1e-325	4	5
Fatty acid metabolism	<1e-325	12	7
Glioma	<1e-325	40	10
Lysine degradation	<1e-325	24	16
Hippo signaling pathway	<1e-325	89	18
ECM-receptor interaction	<1e-325	48	19
TGF-beta signaling pathway	<1e-325	59	22
Signaling pathways regulating pluripotency of stem cells	<1e-325	100	24
Proteoglycans in cancer	<1e-325	128	30
Biosynthesis of unsaturated fatty acids	8.88E-16	9	8
Mucin type O-Glycan biosynthesis	8.65E-11	17	12
Wnt signaling pathway	8.54E-10	43	5
Prion diseases	1.99E-09	2	2
Thyroid hormone signaling pathway	1.56E-08	59	10
Glycosaminoglycan biosynthesis - heparan sulfate / heparin	3.91E-07	9	10
Pathways in cancer	9.90E-07	163	10
Estrogen signaling pathway	2.44E-06	19	6
FoxO signaling pathway	4.67E-06	58	5
Focal adhesion	8.59E-06	54	3
Melanoma	1.84E-05	40	8
Amoebiasis	1.90E-05	24	4
PI3K-Akt signaling pathway	0.0004	74	3
Morphine addiction	0.0005	38	6
Axon guidance	0.0015	61	10
Amphetamine addiction	0.0026	26	6
Prostate cancer	0.0031	16	3

### Protein Characterization in CF Fractions.

**Fig. 7:**
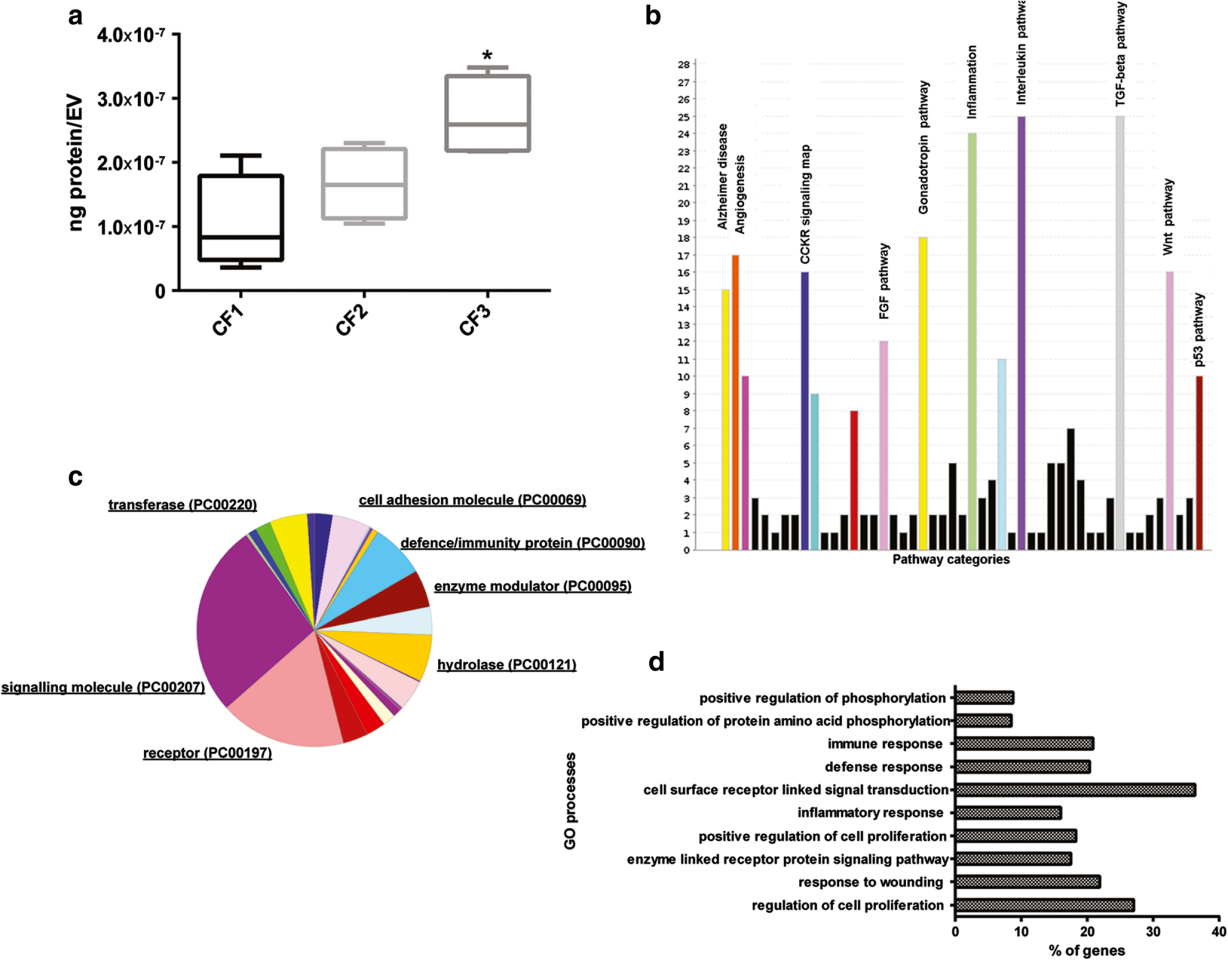
Analysis of CF proteome. The protein composition of the CFs defines their origin and potential activity. (**a**) Quantification of the total proteins isolated from EVs derived from different CFs, demonstrating an enrichment of isolated proteins/EV in CF3 in respect to the other CFs. Data are expressed as means ±SEM (ng protein/EV) of four different experiments. ANOVA with Turkey’s multicomparison test. *P < 0.05 vs CF1. (**b**) Panther pathway analysis on the CF proteome shows abundance of interleukin, inflammation, TGF-β, gonadotropin release hormone receptor, angiogenesis and Wnt signaling pathways. (**c**) Distribution of the 413 proteins compartmentalized in all the CFs in different protein classes. The CF proteome contained the following class of proteins: signaling molecules, receptors, defense/immunity proteins, enzymes and cell adhesion molecules. **d** David GO-BB overrepresented by the CF proteome (*P < 0,001*; FDR 1%). The top ten processes were associated with regulation of cell proliferation, response to wounding, enzyme linked receptor protein signaling pathway, inflammatory response, receptor linked signal transduction, immune response, and regulation of phosphorylation

Protein composition of different CFs could also define their origin and activity. For this reason, proteins were isolated from the three CFs and quantitative proteomic analysis of 1000 proteins was then conducted. Raw data was reported as supplementary information (S3 Table). We observed first a different distribution of total proteins among the three fractions with an enrichment of isolated proteins in respect to the EV quantity in CF3 high-density fraction (Fig. 7a). Less amount of proteins was isolated in CF1 in respect to the high quantity of EVs detected in this fraction.

Proteomic profile of all CFs showed the presence of 655 proteins in the CF2 fraction over the 1000 analyzed. 435 proteins were compartmentalized in the CF3 high-density fraction and 581 in the CF1 low-density fraction. A group of 413 proteins was detected in all the EV populations. Panther pathway analysis on the CF proteome (413 proteins) found high representation of the following pathways: interleukin mediated signaling, inflammation mediated by chemokine and cytokine, TGF-β signaling, gonadotropin release hormone receptor, angiogenesis and Wnt signaling pathways (Fig. 7b). CFs contained proteins which originate from different cellular compartments such as extracellular region, plasma membrane, extracellular matrix, vesicle lumen and secretory granules (Table 4). Moreover, proteins of the CF proteome were mainly incorporated in the following classes: signaling molecules, receptors, defense/immunity proteins, enzyme modulators and cell adhesion molecules (Fig. 7c). The top ten GO biological processes overrepresented by the CF proteome were mainly associated with regulation of cell proliferation, response to wounding, enzyme linked receptor protein signaling pathway, inflammatory response, receptor linked signal transduction, immune response and regulation of phosphorylation (Fig. 7d).

**Table 4:** DAVID analysis to determine GO term enrichment (GOTERM_CC_FAT) for genes coding for proteins of the CF proteome (*n* = 413) (*P < 0,01*; FDR 1%)

GO Term	Count	%	PValue	Bonferroni	FDR
GO:0005615 ~ extracellular space	150	38.66	4.03 E-94	1.04E-91	5.26E-91
GO:0044421 ~ extracellular region part	167	43.04	1.11E-89	2.85E-87	1.45E-86
GO:0005576 ~ extracellular region	226	58.25	4.14E-89	1.06E-86	5.40E-86
GO:0031226 ~ intrinsic to plasma membrane	92	23.71	5.60E-18	1.44E-15	7.31E-15
GO:0005887 ~ integral to plasma membrane	90	23.20	1.41E-17	3.63E-15	1.84E-14
GO:0009986 ~ cell surface	43	11.08	3.29E-15	8.56E-13	4.34E-12
GO:0044459 ~ plasma membrane part	116	29.90	3.79E-11	9.75E-09	4.95E-08
GO:0009897 ~ external side of plasma membrane	24	6.19	7.76E-10	2.00E-07	1.01E-06
GO:0043235 ~ receptor complex	19	4.90	6.19E-09	1.59E-06	8.08E-06
GO:0031012 ~ extracellular matrix	31	7.99	8.64E-08	2.22E-05	1.13E-04
GO:0060205 ~ cytoplasmic membrane-bounded vesicle lumen	11	2.84	3.96E-07	1.02E-04	5.17E-04
GO:0031983 ~ vesicle lumen	11	2.84	6.18E-07	1.59E-04	8.07E-04
GO:0031093 ~ platelet alpha granule lumen	10	2.58	2.09E-06	5.37E-04	0.002
GO:0005578 ~ proteinaceous extracellular matrix	27	6.96	2.32E-06	5.97E-04	0.003
GO:0005886 ~ plasma membrane	152	39.18	2.62E-06	6.74E-04	0.003
GO:0031091 ~ platelet alpha granule	11	2.84	4.17E-06	0.001	0.005
GO:0030141 ~ secretory granule	19	4.90	5.13E-06	0.001	0.006

Differential expression analysis reveals that of the 655 proteins expressed in the medium-density EVs from CF2 fraction, 75 proteins were significantly up-regulated in respect to CF3 and 92 proteins resulted up-regulated in respect to CF1 (Table 5). Of them, 27 were commonly down-regulated in both fractions. Selective proteins were up-regulated in CF1 and CF3 in respect to CF2 fractions (FC CF2 < 0.65) (CF1: *TGFBR2* and *SFRP1* and CF3*: NTRK2, EPHA5, SRMS, LTK, TNFRSF1B, TGFBR1*).

**Table 5:** List of proteins over-expressed in CF2 (FC CF2 > 1.5)

List of proteins over-expressed in CF2 in respect to CF3				
TIMP2	Thymidine Kinase1	BDNF	CCL11	IL1 F6
ALK4	SPINK1	Frizzled6	VEGF R3	Activin B
MCSF R	ALCAM	PlGF	11bHSD1	betaCatenin
hCG alpha	CTGF	IL17RC	TSLP R	TNFRSF12
FGFBP	IL3 R alpha	HGF	Neuropilin2	GPNMB
ACTH	Trypsin 1	IGFI	FGF R4	IL1 R3
PAI1	OSM	CCR8	VWF	CXCL16
VEGFC	CXCR3	CCR6	S100A6	Leptin (OB)
IL21 R	ENA78	IGFBP1	S100A10	CCR1
RAGE	MCSF	TNFRSF1A	TNFRSF11B	IFNalpha / beta R1
Resistin	IL17R	CD56	IL28A	LRP6
GDNF	CXCR2	IL12 p40	Tissue Factor	DANCE
Thrombospondin4	TFPI	proGlucagon	Gastrin	NeuroD1
PLUNC	CCR5	Chem R23	BMP2	CCR2
FGF R3	TOPORS	NT4	TNFSF8	ApoE3
List of proteins over-expressed in CF2 in respect to CF1				
Thrombospondin1	Plasminogen	ICAM5	SRMS	IL15 R alpha
Fibronectin	hCG alpha	NeurokininA	BCAM	BMP2
ApoA4	PTHLP	CD14	CCR6	TNFSF8
Factor XIII A	CNDP1	ALCAM	CCL16	Activin B
Clusterin	APC	CKMB	CD56	betaCatenin
TYRO10	FGFBP	HADHA	HBEGF	IL1 R3
ACK1	CA 153	TNFRSF17	proGlucagon	ICAM2
ALPP	ACTH	TNFRSF5	MMP25	LRP6
Ubiquitin + 1	PAI1	CCR5	ErbB2	DANCE
Btk	Cardiotrophin1	Frizzled5	CCL11	NeuroD1
TNFRSF21	VEGFC	BDNF	Protein p65	IL17C
BAI1	TRKB	APJ	FAP	FGF9
Activin A	CEA	IFNgamma R1	HSP10	CCR2
SERPINA6	CBP	ApoC1	CD80	GLO1
Beta IGH3	IGFBP7	CD117	TNFSF3	PDX1
CA 125	Calcitonin	IGFI	ErbB3	ApoE3
ABL1	AR	IL29	EGF	
TIMP2	Thymidine Kinase1	CCR8	Apelin	
ALK4	C9	IL1 F5	ADAMTS10	

**Fig. 8:**
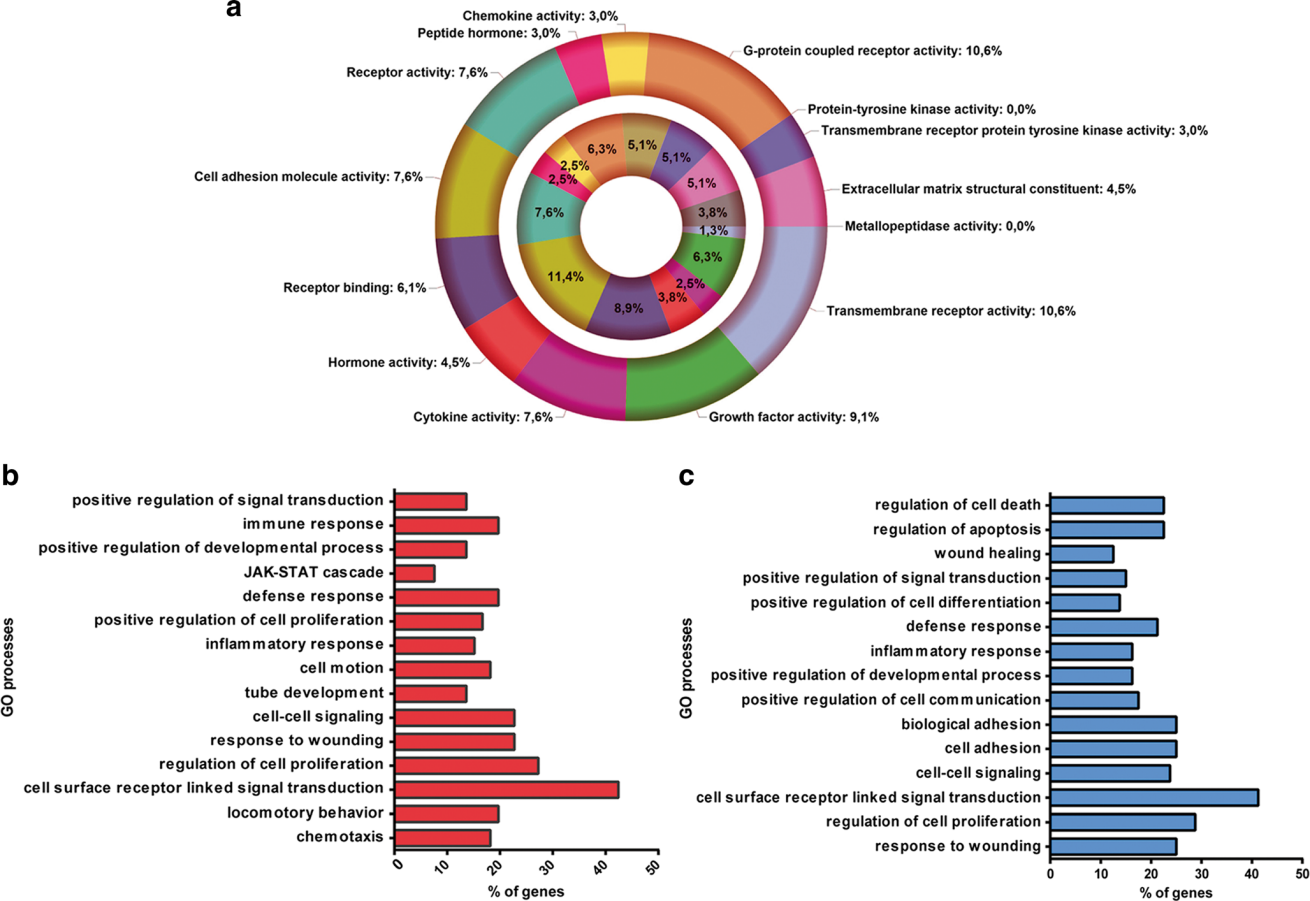
Differential expression analysis of proteins in EVs from different CF fractions. **a** David GO-MF terms over-represented by the proteins upregulated in the medium density CF2 in respect to the CF3 high density fraction (*n* = 75, FC CF2 > 1.5, outer chart). The same analysis was conducted for proteins upregulated in CF2 in respect to the low-density fraction CF1 (*n* = 92, FC CF2 > 1.5, inner chart). (B-C) Distribution analysis of the proteins enriched in CF2 in respect to CF3 **b** and to CF1 **c**, in different biological processes (GO-BB). The main representative classes were: signal transduction via receptor interaction, cell proliferation, response to wounding, cell-cell signaling and inflammatory response. (*P-value < 0,01*, FDR 1%)

To investigate the differences at biological levels among fractions, we performed functional enrichment analyses of the proteins upregulated in CF2 in respect to CF3 and CF1. Enriched GO molecular function (GO-MF) terms over-represented by the proteins upregulated in CF2 in respect to CF3, were mainly incorporated in: transmembrane receptor and receptor-binding activities, G-protein couple receptor activity, cytokine and hormone activities and cell adhesion molecules (Fig. 8a, outer chart). The GO-MF overrepresented for proteins upregulated in CF2 in respect to the low-density fraction CF1 were largely associated with cell adhesion molecules, receptor binding and activity (Fig. 8a, inner chart). Selective molecular functions were metallopeptidases and protein tyrosine-kinase activity (Fig. 8a, inner chart).

Enriched GO biological processes (GO-BP) of proteins upregulated in CF2 in respect to CF3 were chemotaxis, signal transduction via receptor interaction, cell proliferation, response to wounding, cell-cell signaling, development and inflammation (Fig. 8b). Enriched GO-BP were also observed, for proteins down-regulated in CF1 in respect to CF2. These include common processes such as signal transduction via receptor interaction, cell proliferation, response to wounding and cell-cell signaling. Selective processes such as cell adhesion, regulation of cell differentiation and apoptosis were also detected (Fig. 8c).

## Discussion

The results of the present study demonstrated that EVs isolated from the conditioned medium of MSCs, using the gradient separation technique, are heterogeneous in their quantity and composition. Based on the differential expression of specific exosome enriched-markers and different density among the twelve fractions, we grouped them in three combined fractions. The combined fractions, displayed differential pro-proliferative and anti-apoptotic activities on renal tubular epithelial cells. Comparative miRNome and proteomic profiles, revealed a cluster of miRNAs and proteins common to the three vesicle fractions and fraction specific subsets of RNAs and proteins.

EVs have been described as important players of the MSC secretome. In fact, the beneficial effect of MSC treatment on different models of acute and chronic damages was mimic by a vesicles-based therapy [2, 16, 34–36]. EVs are composed of several subpopulations and most of their characteristics are not exclusive of a specific class of EVs [23]. For this reason, the possibility that they act as distinct biological entities is now under evaluation [37].

EVs released by MSCs, were a mixed population with different diameters and with two prominent peaks around 100 and 180 nm, as detected by NTA. EVs isolated by discontinuous density gradient separation showed a heterogeneity in quantity and expression of the classical exosomal markers. The exosomal markers were mainly detected in the medium-density gradient fractions (1.08–1.14 g/mL density) which co-expressed the CD63 with classical mesenchymal vesicular markers such as CD29 and ITGA5 [2], and ANXA2 an important effector of miRNA recruitment in EVs [38].

The selective expression of miR-451, in the central fractions of the gradient EVs, further supports the enrichment in exosomes of this fraction, since previous studies in tumor cell lines showed that this miRNA is preferentially sorted into exosomes [39].

According to Xu et al. [40], the denser fractions contained microvesicle-enriched populations. CF3 denser fraction, despite the presence of CD63, does not or barely express the HLA-1 and ITGA5 suggesting that is a different population from CF2. Moreover, CF3 is enriched in EVs of larger size (>200 nm) and showed the highest mean diameter among the different EV populations, supporting the enrichment in microvesicles of this fraction. The low-density CF1 fraction contained an undefined population of EVs, that expressed low levels of CD29 and ANXA2 and were negative for all the other markers tested.

Xu et al. demonstrated that exosomes and microvesicles isolated by differential centrifugation from human colon cancer cells, have distinct biological activities, being able to promote invasiveness at different rating [40]. Moreover, Aliotta et al. showed that exosomes-enriched and microvesicles-enriched populations from mouse MSCs, have different effects when infused into mice, in a model of monocrotaline induced pulmonary hypertension [41]. The same biological differences in mouse and human MSC EVs sub-populations were demonstrated by Wen et al., using a model of marrow radiation damage [42]. We previously showed the ability of MSC EVs, to promote proliferation and to protect from apoptosis murine and human renal epithelial tubular cells [2, 43]. These effects were ascribed to a heterogeneous population of EVs containing both microvesicles and exosomes. We here demonstrated that different EV fractions separated by discontinuous iodixanol gradient based on their density, display differential pro-proliferative and anti-apoptotic activities on recipient renal tubular cells despite they were equally internalized by mTEC. EVs from CF1 and CF2 low and medium-density fractions induced proliferation on renal tubular cells, cultured in the absence of serum. However, the CF2 enriched-exosome population was more effective than the others combined fractions in the protection from apoptosis induced by hypoxia/reperfusion injury on renal target cells. High-density EVs in fraction CF3, conceivable containing a microvesicle-enriched population, did not induce significant protection from apoptosis and proliferation of renal tubular cells, also after enhancing the dose of EVs administered.

The activity of EVs has been at least in part ascribed to their miRNA content [42–45]. A subset of miRNA families, was specific signature of the biologically active CF2 medium-density fraction. Some of these miRNAs, such as miR-17-5p and miR-106a have been described together with miR-21 as differentially expressed during different phases of renal injury, being potential biomarkers for AKI [32]. Interestingly, different miRNA* sequences were only detected in CF2 fraction, supporting the potential of exosomes to transfer miRNA* outside the cells [14].

Several other miRNAs enriched in CF2 fraction, were reported as protective in the context of AKI including miR-34 [27], miR-125b [31], miR-199a-3p, miR-214 [28] and miR-127 [29]. miR-21 and miR-29, enhanced in CF2, have been linked to the modulation of kidney fibrosis [30]. miR-451 selectively present in the central fractions of the gradient, has been demonstrated to act together with miR-144, in the protection against IRI in the heart [46]. All the miRNAs analyzed were reduced in the CF1, supporting a less enrichment of miRNAs in this fraction.

Enrichment analysis of the pathways over-represented by the predicted targets of enriched/selective CF2 miRNAs showed strong correlation with metabolic, stem cell associated- and migration/inflammation processes. High representation of fatty acid biosynthesis and metabolism, Wnt signaling and pluripotent stem cells regulated-pathways, ECM-receptor interaction and TGF-β signaling pathway have been detected. Interestingly, downregulation of fatty acid oxidation in tubular cells has been described as a key component of the pathogenesis of AKI [47]. Moreover, stem cell and Wnt/β-catenin signaling pathways have been also correlated with repair processes after ischemic AKI [48].

Proteins are together with RNA, important effectors for the EV activity. By protein array, we identified a protein cargo of 413 proteins detected in all the different EV fractions. Cross match with Vesiclepedia database [49] identified 205 classical EV proteins detected in all the EV fractions. Proteins of the CF proteome were mainly incorporated as signaling molecules, receptors and cell adhesion molecules, recently connected with the potential therapeutic effects of the microvesicles from MSCs [50]. Defense/immunity proteins and enzyme modulators class of proteins were also detected in the CF proteome. Moreover, they were classified as extracellular, plasma membrane, matrix and vesicle proteins. Pathway analysis of the CF proteome reveals high representation of pathways such as interleukin mediated signaling and chemokine/cytokine mediated inflammation, Wnt signaling, TGF-β and angiogenesis pathways. MSC CF proteome was relevantly enriched in anti- and pro-inflammatory cytokines.

Moreover, EVs from all CF fractions expressed the chemokines receptors of cell of origin, such as CXCR1, CXCR6, CXCR4, CXCR3, CCR3 and CCR7, known to be involved in the MSC migration to the sites of inflammation [51]. Eph A4 and different subclasses of the Eph B receptors, regulators of MSC attachment/migration [52] and inhibitors of T cell proliferation [53], were also present in all EV fractions. Moreover, numerous pro-angiogenic and pro-migratory molecules such as the soluble factors VEGF, TGF-β, ΙL-8 and PDGF and PDGFRα/β were compartmentalized in all the CF fractions. Interestingly, increasing angiogenesis together with renal blood flow have been recently observed in IRI rats treated with adipose mesenchymal stem cell exosomes and associated with the reversion of the kidney damage [54]. The catalytic subunit PI3-Kinase p85-beta, detected among the 50 major expressed proteins of CF proteome, has been recently correlated with cell cycle re-entry and proliferation via PI3K/Akt signaling pathway activation [55].

The protein associated biological processes overrepresented in CF2, which was the most biologically active fraction, included positive regulators of proliferation, response to wounding and cell-cell signaling possibly accounting for the pro-proliferative activity of CF EVs. Migration/proliferation processes were also over-represented by the enriched miRNAs in the medium-density CF2 fraction, showing a collaborative pattern of proteins and miRNAs shuttled by MSCs in promoting regenerative processes. Interestingly, TGF-β pathway crucial in AKI progression to chronic kidney disease [56] was overrepresented both by miRNAs and proteins compartmentalized in the CF2 fraction, suggesting the regulation of this pathway by MSC EVs treatment.

Of interest, the major cytokine enriched in CF2 fraction was the IL-13 that together with IL-10 and IL-4, mediates differentiation of monocytes in non-inflammatory (M2) macrophages [57]. Recently, MSCs have shown the ability to educate macrophages to acquire an anti-inflammatory M2 phenotype, promoting kidney repair in rhabdomyolysis-induced acute kidney injury [58]. Further, CF2 EVs contained high levels of ApoC3 and ApoA4, recently detected inside the EVs produced by umbilical cord blood-derived MSCs with other family members and involved in inflammation and tissue repair [59].

In conclusion, this study demonstrated that EVs derived from MSCs are heterogeneous with specific signatures accounting for the biological activity of different EV populations. Proteins and miRNAs shuttled by MSCs showed a collaborative pattern in crucial processes activated after injury, such as metabolic, stem cell, inflammation/migration and angiogenic related processes. Moreover, the medium-density CF2 fraction containing exosome-enriched population of EVs showed the best activity in promoting renal protection from injury in vitro. This fraction was enriched in miRNAs and proteins associated with biological processes fundamental in kidney regeneration.

## Electronic supplementary material

ESM 1 (DOCX 13 kb)

ESM 2 (DOCX 13 kb)

ESM 3 (XLSX 162 kb)
