# Supplementary material for: Exosome and Microvesicle-Enriched Fractions Isolated from Mesenchymal Stem Cells by Gradient Separation Showed Different Molecular Signatures and Functions on Renal Tubular Epithelial Cells
Source: Stem Cell Rev. 2017 Jan 9;13(2):226–43. doi: 10.1007/s12015-016-9713-1 (PMC5380712; doi:10.1007/s12015-016-9713-1)
Supplement: Supplementary file 1 — (DOCX 13 kb) [file 12015_2016_9713_MOESM1_ESM.docx]

**Supplementary Table 1.** Biological pathways over-represented by the miRNAs enriched in the medium dense CF2 fraction in respect to CF3 high dense fraction (n=89 miRNAs) (*P<0.01*, FDR corrected).

| **KEGG pathway** | **p-value** | **#genes** | **#miRNAs** |
| --- | --- | --- | --- |
| Fatty acid biosynthesis | <1e-325 | 4 | 3 |
| Prion diseases | <1e-325 | 2 | 5 |
| Signaling pathways regulating pluripotency of stem cells | <1e-325 | 77 | 10 |
| ECM-receptor interaction | <1e-325 | 43 | 11 |
| Mucin type O-Glycan biosynthesis | <1e-325 | 21 | 14 |
| TGF-beta signaling pathway | 1.14E-14 | 46 | 11 |
| Glioma | 4.54E-11 | 34 | 8 |
| Morphine addiction | 2.92E-10 | 42 | 9 |
| Proteoglycans in cancer | 3.19E-10 | 105 | 15 |
| Hippo signaling pathway | 9.14E-05 | 70 | 10 |
| Lysine degradation | 0.0009 | 19 | 8 |
| Amphetamine addiction | 0.0023 | 32 | 8 |
| FoxO signaling pathway | 0.0024 | 62 | 7 |
| Glycosaminoglycan biosynthesis - heparan sulfate / heparin | 0.0092 | 8 | 9 |
