# Supplementary material for: Exosome and Microvesicle-Enriched Fractions Isolated from Mesenchymal Stem Cells by Gradient Separation Showed Different Molecular Signatures and Functions on Renal Tubular Epithelial Cells
Source: Stem Cell Rev. 2017 Jan 9;13(2):226–43. doi: 10.1007/s12015-016-9713-1 (PMC5380712; doi:10.1007/s12015-016-9713-1)
Supplement: Supplementary file 2 — (DOCX 13 kb) [file 12015_2016_9713_MOESM2_ESM.docx]

**Supplementary Table 2.** Biological pathways over-represented by the miRNAs selective present in the medium dense CF2 fraction in respect to CF3 high dense fraction (n=97 miRNAs) (*P<0.01*, FDR corrected).

| **KEGG pathway** | **p-value** | **#genes** | **#miRNAs** |
| --- | --- | --- | --- |
| Signaling pathways regulating pluripotency of stem cells | 1.48E-10 | 90 | 14 |
| Proteoglycans in cancer | 2.14E-08 | 108 | 15 |
| Lysine degradation | 4.12E-08 | 13 | 8 |
| TGF-beta signaling pathway | 2.61E-07 | 52 | 11 |
| Biosynthesis of unsaturated fatty acids | 1.17E-06 | 9 | 8 |
| Fatty acid metabolism | 1.82E-05 | 9 | 7 |
| Hippo signaling pathway | 0.0002 | 60 | 8 |
| ECM-receptor interaction | 0.0004 | 28 | 9 |
| Mucin type O-Glycan biosynthesis | 0.0006 | 14 | 10 |
| Fatty acid biosynthesis | 0.0022 | 1 | 2 |
| Estrogen signaling pathway | 0.0095 | 20 | 7 |
